# Using Goal-Directed Design to Create a Novel System for Improving Chronic Illness Care

**DOI:** 10.2196/resprot.2749

**Published:** 2013-10-29

**Authors:** David Fore, Linda M Goldenhar, Peter A Margolis, Michael Seid

**Affiliations:** ^1^LybbaLos Angeles, CAUnited States; ^2^Cincinnati Children’s Hospital Medical CenterThe James M Anderson Center for Health Systems ExcellenceCincinnati, OHUnited States; ^3^Cincinnati Children’s Hospital Medical CenterPulmonary MedicineThe James M Anderson Center for Health Systems ExcellenceCincinnati, OHUnited States

**Keywords:** goal-directed design, learning health care system, chronic illness care, health system design, quality improvement, pediatrics

## Abstract

**Background:**

A learning health system enables patients, clinicians, and researchers to work together to choose care based on the best evidence, drive discovery as a natural outgrowth of patient care, and ensure innovation, quality, safety, and value in health care; all in a more real-time fashion.

**Objective:**

Our paper describes how goal-directed design (GDD) methods were employed to understand the context and goals of potential participants in such a system as part of a design process to translate the concept of a learning health system into a prototype collaborative chronic care network (C3N), specifically for pediatric inflammatory bowel disease.

**Methods:**

Thirty-six one-on-one in-depth interviews and observations were conducted with patients (10/36, 28%), caregivers (10/36, 28%), physicians/researchers (10/36, 28%), and nurses (6/36, 17%) from a pediatric gastroenterology center participating in the ImproveCareNow network. GDD methods were used to determine the context and goals of participants. These same methods were used in conjunction with idealized design process techniques to help determine characteristics of a learning health system for this pediatric health care ecology. Research was conducted in a clinic and, in the case of some patients and caregivers, at home.

**Results:**

Thematic analysis revealed 3 parent-child dyad personas (ie, representations of interviewees’ behavior patterns, goals, skills, attitudes, and contextual information) that represented adaptation to a chronic illness over time. These were used as part of a design process to generate scenarios (potential interactions between personas and the learning health system under design) from which system requirements were derived. These scenarios in turn helped guide generation, prioritization, design, measurement, and implementation of approximately 100 prototype interventions consistent with the aim of C3N becoming a learning health network.

**Conclusions:**

GDD methods help ensure human goals and contexts inform the design of a network of health care interventions which reflect the shape and purpose of a C3N in pediatric chronic illness care. Developing online and in-person interventions according to well-documented context and motivations of participants increases the likelihood that a C3N will enable all participants to act in ways that achieve their goals with grace and dignity. GDD methods complemented quality-improvement methods to generate prototypes consistent with clinical and research aims, as well as the goals of patient disease management.

## Introduction

### Problem Statement

Our current health care system is far too costly, with less than impressive returns in health. The Institute of Medicine (IOM) considers this a systemic issue rather than one owing to any particular set of isolated factors. As such, the IOM champions “learning health systems that enable patients and providers to work together to choose care based on the best evidence, drive discovery as a natural outgrowth of patient care, and ensure innovation, quality, safety, and value in healthcare; all in a more real-time fashion” [1]. Despite the appeal of such a system, there are only a few specific instances of learning health systems [2,3].

Instantiating the vision of a learning health system requires a systematic method for translating a promising concept into actual tools and processes that work together as new characteristics of existing systems of care. Understanding the needs of potential participants in a learning health system is central to generating new ideas that will lead to a fundamental redesign to better meet the needs of patients, parents, clinicians, and researchers alike. Meanwhile, communication and information technologies are seen as key to success of a learning health system; indeed a significant majority of patients and their advocates undertake Internet-enabled health-seeking and health-making behaviors [4]. However, these technologies too often fail to live up to their promise [5-10]. Only a minority of software project initiatives succeed, the disappointing numbers largely owing to difficulties in accurately ascertaining requirements in advance of design and construction [11].

Kano et al [12] identified 3 different types of participant needs: (1) revealed needs (wants) are typically achieved by just asking people what they want, (2) expected needs are often so basic that people may fail to mention them until a service or product fails to perform them, resulting in dissatisfaction, and (3) exciting needs, which are difficult to discover, are unspoken, and expand the customer’s expectations.

### Product Research and Design Methods

Goal-directed design (GDD) is a research-based software-design method for anticipating how people will respond to a new or modified product, service, or system [13]. GDD also precisely articulates the shape and purpose of online and offline system elements that will help people meet their goals. GDD is employed at the outset of the product-definition process so as to define human requirements of a system before design and construction take place. GDD’s synthesis and design methods guide generation, communication, and specification of products to the larger team.

Human-computer interaction (HCI) methods are used to facilitate more productive interactions between people and software programs [14,15]. User-centered design (UCD) emerged in response to HCI by situating humans at the center of product-development process through “user research” and other techniques that reveal responses people have to an existing product to which they are exposed [16]. UCD’s application in health care has included designing and developing patient monitors [17], clinical decision-making tools used in clinical situations [18], and patient health technologies for the aging population [19]. It is often restricted to involving potential participants after the product is developed [20,21].

GDD is an outgrowth of HCI and UCD. GDD explicitly acknowledges that there are no “users” of a product not yet designed; so, instead, GDD offers techniques (personas, scenarios, interaction-design patterns, and principles) for positing a future where a new or modified product exists to satisfy human goals. Other research and synthesis methods such as contextual inquiry [22] emphasize a cross-disciplinary team-based approach to determining needs and opinions of “customers” (ie, purchasers) largely through ethnographic methods, analysis, and synthesis techniques performed by large teams of software developers, product managers, designers, and others. GDD methods hinge on the observation that software is infrequently used by purchasers and so offers methods for determining the needs and context of people likely to use the software and those who will be effected by somebody else’s use of software; teams are smaller and the process is far less time-consuming (ie, several weeks vs several months).

GDD serves the inherently subjunctive nature of the design process by distinguishing goals from tasks, thereby equipping designers with means to design what will satisfy goals of future participants through alternate and possibly superior means than the current means. GDD begins with ethnographic and observational methods that help a team understand what is required of a new system built to engage and satisfy those for whom it is intended. GDD uses fictional composites of potential participants of the new system, called “personas,” which are defined according to their contexts, capabilities, and goals. Designers then create “scenarios” that describe specific interactions that may occur between personas and the system, from the beginning of a task through goal achievement, keeping in mind the personas’ contexts and motivations. Creating a set of personas and scenarios serves several purposes. Doing so helps identify and communicate critical characteristics of both the technical and human parts of a system featuring which specific innovations. This method takes into account how people will likely interact with the system; what aspects of the new system are worth the design, development, and testing of potential solutions; and how interactions can be re-designed to achieve desired behavior while balancing the contexts and needs of all concerned. This diminishes a design team’s temptation to project their own needs onto system participants, curbs proliferation of unnecessary features, and prevents centering the system design around “edge cases” or outliers [23]. Finally, personas and scenarios serve to inform detailed design specifications for constructing the online and complementary offline processes.

### Objectives

Our paper provides an illustration of how GDD methods were used as part of an effort to translate the IOM’s vision of a learning health system for chronic care management into a prototype system that we call a collaborative chronic care network (C3N). The aim is to demonstrate that GDD methods can yield a design of a learning health system to which participants will respond favorably.

## Methods

### Setting

The ImproveCareNow network [24] is a multisite practice network of pediatric gastroenterology practices treating children and youth with inflammatory bowel disease (IBD; Crohn’s disease and ulcerative colitis [UC]). ImproveCareNow was formed with support of the American Board of Pediatrics and the North American Society for Pediatric Gastroenterology, Hepatology, and Nutrition as the pilot program for development of performance-based maintenance of board certification [25,26]. ImproveCareNow sites have population-based registries with common data elements, share process and outcomes data, and use quality improvement methods to make changes in care delivery. Through this process of clinician-led collaboration and standardization of care, the network has dramatically increased the proportion of patients in remission [27].

### Participants

Patient, parent, and nurse participants were recruited from Cincinnati Children’s Hospital Medical Center (CCHMC), one of the pediatric gastroenterology centers participating in the ImproveCareNow network. Physicians/researchers were recruited from CCHMC, the University of North Carolina at Chapel Hill, the Children’s Hospital of Philadelphia, Children’s Hospital, Oakland, and Dell Children’s Hospital of Central Texas, in Austin. Adolescent/young adult patients (age, 12-22 years) with IBD and their parents/caregivers were recruited by their physicians or responded to fliers posted in the IBD clinic. Interested patients and parents were contacted, provided with further explanation of the study, and asked for a convenient time to schedule the interview. This study was approved by the CCHMC Institutional Review Board (IRB).

### Data Collection

In-depth, face-to-face interviews and direct observations were conducted in January and February 2010. The topics in the semi-structured interview guide were designed to obtain an in-depth understanding of the participant’s expressed and observed contexts, goals, and attitudes related to having and managing a chronic disease in general and IBD specifically [28]. Interviews were conducted by 2 of 3 experienced interaction designers with training in GDD methods. One designer led the interview while the other took notes and probed to ensure complete understanding of the answers provided. Interviews took place in a private room at the hospital, the interviewee’s home, another location as desired by the parent, or by phone or video conferencing (in the case of researchers). Patients and their parents were interviewed separately to reduce undue influence on responses. Sample topics for the patient/parent interview are provided in Textbox 1. While each topic was discussed with each subject the sessions were conducted in a conversational style that permitted interviewees to express themselves extemporaneously. Interviews lasted 1.5 hours, were audio-taped, then transcribed verbatim. Families were offered a US $20 gift card for participation. Observations took place in the IBD clinic, where one designer observed and took notes, shadowing patients/parents and clinicians. Observations also took place at a network “learning session” where participating care sites met to share methods to improve care.

**Sample of semi-structured interview questions.**

1. Tell me what it is like to manage your disease.

2. Walk me through a typical day, like yesterday.

   (a) A particularly difficult day? A carefree day?

   (b) What are the hardest parts?

3. What works well for you in terms of managing your disease?

4. If you were to describe to someone who had no idea about Crohn’s or ulcerative colitis, or what it’s like living with it, what would you say?

5. If you could change one thing about what you have to do to manage your disease, what would it be?

6. Do you have friends who also have Crohn’s or ulcerative colitis? Tell me what that is like.

7. Do you ever use the Internet to get information about Crohn’s or ulcerative colitis? In what way does this help you?

   (a) Do you ever use the Internet to connect with other kids with Crohn’s or ulcerative colitis? Tell me more about that.

   (b) Can you tell me about the kinds of things would you like on the Internet that would be useful to you, but you’ve not been able to find?

### Analysis

Transcripts were coded with an identification number then de-identified. Notes from each session were reviewed and incorporated into the transcripts if they provided additional information. All three team members analyzed the patient and parent transcripts independently and analyzed, synthesized, and categorized overarching common themes and subthemes based on similar characteristics, context, and goals. The themes were incorporated into a spreadsheet and given a color code that allowed for pattern recognition. The respondents’ comments were categorized within themes and subthemes that emerged from the data. Patterns of comments within and across theme/subthemes were identified to assess goal-related relationships across interviewees. Differences in coder opinion were discussed and agreement was achieved before final decisions on existing patterns were made. The identified patterns were used to create the personas and scenarios. The personas and scenarios were shared with a subset of the original sample of patients, parents, clinicians, and researchers. Qualitative feedback was elicited regarding the extent to which these represented the participants’ goals, contexts, and capabilities and were illustrative of potential interactions in a new system.

## Results

### Overview

The demographic characteristics of IBD patients and their parents are presented in Table 1. The 3 male and 7 female patients ranged from 14 to 22 years of age; 5 had a diagnosis of Crohn’s disease and 5 had UC. The 8 male and 3 female physicians/researchers ranged from 33 to 55 years of age and had 3-31 years of clinical experience. The 6 nurses (all female) ranged from 36 to 60 years of age and had 7-30 years of clinical experience. Patients included 5 with Crohn's disease and 5 with UC. Three were less than 2 years post-diagnosis, while the rest were more than 5 years post-diagnosis. Daily regimens ranged from 1 to 40 pills, and the range of surgeries per patient ranged from 0 to 3. Comorbidities included juvenile idiopathic arthritis, asthma, primary sclerosing cholangitis, autoimmune hepatitis, attention-deficit hyperactivity disorder, depression, and allergies. For parents, 8 were married, with 2 divorced, 5 were employed full-time, 2 part-time, and 3 unemployed. Educational attainment ranged from no high school (1/10), to high school (4/10), to college graduate (5/10).

### Themes

The strongest themes that emerged around patients included time since diagnosis, symptom control, degree of reliance on parents for treatment decisions, and degree of isolation or connection with others with the disease. Predominant emergent parent themes consistently reflected their child’s condition: time since diagnosis, child’s level of coping, and experience with chronic disease. In fact, the differences among parents had less to do with their own personal psychographic characteristics and almost everything to do with their child’s dimensions and themes. Demographic characteristics (eg, ethnicity, income, and gender) were analyzed for salience and found to be insignificant compared with the aforementioned themes. Themes were not predetermined but rather emerged from patterns in the research, and coalesced into 3 patterns as illustrated in Table 2.

### Personas

Because the themes were so similar, in this sample, between parents and patients, we created familial dyad narrative descriptions for personas. Given that 3 patterns emerged from the interviews, we developed 3 dyads. These dyads were: Orleans (daughter)-Floyd (father), Bianca (daughter)-Bram (father), and Uri (son)-Jody (mother). They vary on demographic characteristics, the degree to which they understand disease processes, adherence, and self-management behaviors, and, most importantly from the design perspective, their disease-related goals. Table 3 presents personas for Orleans and Floyd. Other personas, including those of a physician, a nurse, and a researcher, are available in Multimedia Appendix 1.

**Table 1:** Demographic characteristics of ImproveCareNow IBD patients and their parents (N=20).

		Patients, n=10	Parents, n=10
**Age (years)**			
	Range	14-22	35-55
	Mean (SD)	18 (3)	42 (6)
**Gender, n(%)**			
	Males	3 (30)	3 (30)
	Females	7 (70)	7 (70)
**Race, n (%)**			
	White	7 (70)	8 (80)
	Black	2 (20)	1 (10)
	Other	1 (10)	1 (10)

**Table 2:** Examples of patient and parent themes used to create personas.

	Patients			Parents		
	Pattern 1	Pattern 2	Pattern 3	Pattern 1	Pattern 2	Pattern 3
**Themes and characteristics**						
	Diagnosed recently to 2 years ago	Diagnosed 5+ years ago	Diagnosed >5 years ago	Child was diagnosed 2 or fewer years ago	Child diagnosed more than 2 years ago	Child was relatively old when diagnosed
	On meds, symptoms not under control	Symptoms under control	Complicated medical history	None to very little experience with chronic conditions	Experience with chronic disorders	Child now a young adult
	Relies on parents and practitioners for treatment decisions	Relies on parents for managing care, taking more responsibility	Transitioned into adult care	Feels overwhelmed by the child’s disease	Taking the child’s disease in stride	
			Symptoms under control			
			Care and treatment are driven by personal preferences			
			Involved with IBD communities			
**Goals**						
	Control symptoms	Feel normal	Keep symptoms controlled	Make sure child is independent and adherent, charts pills, and keeps appointments	Reduce stress by reducing overall burdens of care	Manage stress particularly during patient’s flare-ups
	Find community	Get on with life	Be a leader			
	Be understood					

**Table 3:** Illustrative personas.

	Persona	Goals	Characteristics
**Orleans—Patient**			
	Age 13, African-American, and diagnosed 2 years ago with UC and autoimmune hepatitis	Control symptoms	Still getting her bearings Flares are frequent Often on prednisone, which makes her look and feel unusual
		Be understood	Struggles with dad on how to care for herself Has to take many pills that do not always seem to work Recently diagnosed with depression
			Hard to watch what she eats, particularly when she is with other kids Socially isolated, repeatedly absent from school, trusts only a few friends Tries to stay active, practices yoga, and plays basketball when she is feeling well Uses the Internet for playing games, downloading music, email, and MySpace Leaves the Internet IBD research to her dad
		Illustrative quote: “I like to play video games.”	
**Floyd Jackson—Orleans’ Dad**			
	Age 38, married, 3 children, and self-employed auto repair shop owner	Make the right choices	Has no experience with chronic disease Orlean’s primary caretaker: makes appointments, picks up prescriptions, and communicates with the nurse Is concerned about paying for Orleans’ ongoing treatment, especially if she gets worse and his wife has to stop working to care for her
		Keep track of it all	Skeptical about medications, has heard about some long-term side effects Is interested in nonpharmaceutical therapies Seeks advice from many sources, including friends and websites
		Find a healing community	Worried that Orleans is becoming irritable and solitary and that she is depressed
		Maintain financial stability	
		Illustrative quote: “It would be good for her if she knew someone else with ulcerative colitis.”	

### Scenarios

Scenarios started from 2 design premises: (1) better-informed and more productive interactions (both online and offline in face-to-face or phone interactions) between and among patients, parents, and clinicians will result in improved health outcomes and (2) systems that encourage open and safe access to people and information are necessary for facilitating these interactions. The scenarios aimed at enabling a persona to achieve personal goals starting from the premises stated above and also be based on realistic technical possibilities of an online and face-to-face system that could be developed. Pediatric patients from whom personas were derived strongly suggested that scenarios should also provide feedback that measures progress, incorporate small tasks that encourage learning, establish a community to support public commitments, and make interaction engaging and fun.

The personas’ context, disease state, goals, and other characteristics were used in a scenario-generating process (which also included environmental scanning, review of the literature, role playing, and input from clinicians and others who comprise C3N) to develop scenarios describing potentially viable and desirable ways a new C3N system and its potential participants would interact with about 80 prototypes. One example of a scenario supporting a prototype (“personal experiment” or N=1 trial) is presented in Textbox 2 for the personas of Orleans and her father, Floyd.

Feedback from participants indicated that the personas and scenarios accurately captured the goals and contexts of participants and realistically portrayed potential experiences of patients and parents with a new health care system. These scenarios, in turn, drove detailed requirements that contributed to detailed descriptions for prototypes of digital health care interventions; ultimately the scenarios drove the design, construction, and operation of a learning health network which deploys such interventions. Further example scenarios and design screenshots are available in Multimedia Appendix 2.

**Scenario 1: Orleans and Floyd—Pursuing a personal experiment trial.**

1. Because of Floyd’s interest in Orleans safely experimenting with nonpharmaceutical approaches to managing her symptoms, he asks Dr Roan, at Orleans’ next appointment, about probiotics for her cramps and diarrhea.

2. Dr Roan talks with Orleans and Floyd about a personal experiment trial to test whether probiotics will work. Together they decide to begin a personal experiment to see how she responds to over-the-counter probiotics while remaining on her current course of treatment.

3. Dr Roan opens the online C3N Portal and links to the template for a personalized experiment trial. She selects the type of probiotics they will use and, since Orleans has a phone that can text-message, uses the SMS survey function to create 2 daily SMS messages for Orleans to (1) remind her to consume the probiotics and her current meds and (2) assess the 2 symptoms in question.

4. The personal experiment trial system suggests three 2-week experimental periods: 2 weeks on probiotics, 2 weeks off, and 2 weeks on again.

5. Invitation to and information about the trial is sent to the email services used by Orleans and because of the personal settings Floyd also gets the email. They watch a short video explaining what personal experiment trials are about and complete an online survey about her health and views. The system confirms the submission to all parties and provides next steps for each to follow.

6. At the same time, the personal experiment system accurately identifies Orleans and protects her privacy and that of her father while ensuring adherence with other elements of the IRB protocol.

7. As Orleans shares observations of her health the system stores data in such a way that Orleans and her father have continuous access to it for their own use, and so that they can offer clinicians and researchers permission to access and use those data for Orlean’s benefit and for population studies.

8. In collaboration with her doctor, Orleans and her father learn to observe and share health-related behaviors with one another and with Dr Roan, and learn to modify those behaviors based upon correlations Dr Roan identifies between behaviors and Orleans symptoms and her clinical data.

### System Requirements for Prototypes

The chief reason to employ GDD methods is to arrive at content, data, technical, scientific, and regulatory requirements for the purpose of furthering the goals of multiple participants in the health care ecology simultaneously. The scenario described in Textbox 2 yields in-person and online design requirements for a prototype self-experimentation system that centers around the patient (Orleans) and her caregiver (Floyd), with her clinician (Dr Roan) and researchers playing supporting roles. These included:

- An online clinician portal to craft and share experiments with others.
- Templates for experiments.
- Ability for clinician to revise templates for particular patient needs and to submit revisions to system.
- Ability for others to use the revised templates.
- Patient and caregiver access to experiment templates prepared by clinician.
- Mobile phone tools that make it natural for a teenager to participate in her own personal experiment.
- Media (videos, etc) that a pediatric patient and her parents will find compelling and which clinicians judge consistent with best medical guidance.
- A survey function for collecting baseline, ongoing, and closing observations of patients and caregivers for personal use and for purposes of population studies.
- System for unique identification that protects the privacy of patients and caregivers while ensuring IRB adherence.
- Data storage system that provides access to patients and caregiver for their own use, and allows them to provide conditional permission to clinicians and researchers to access those data.

## Discussion

### Themes

We envision a C3N as one way to create a learning health system, but translating the concept of a learning health system to an actual one requires a systematic process for identifying patterns of participant goals and realistic ways that patients, researchers, and clinicians might interact within the newly constructed ecosystem. Using GDD methods, we collected and synthesized a range of critical qualitative data from stakeholder representatives about their goals and values. We identified 3 major segments of patient participants, as well as 3 clinician/researcher participants, differentiated by an understanding of needs and goals. We then developed scenarios to help guide the design of a network of prototypes that comprise C3N. These prototypes are now in use in the C3N population.

Several themes common in the literature of pediatric care are not directly addressed in this paper even where such themes arose in our research. For example, the theme of independence and the related theme of transition to adult care characterize both “Pattern 2” and “Pattern 3” the patient/parent dyads depicting an older teen and a young adult, respectively. In interest of space, however, the authors draw our examples exclusively from “Pattern 1” (Orleans and Floyd); this pattern merits focus owing to intense challenges associated with a patient/parent dyad with a relatively recent diagnosis of a serious chronic condition, and the consequent needs they have for well-designed interventions that will help forge richer connections between patient and caregivers.

### Literature

Although the medical and public health literature contains numerous studies describing the use of qualitative methods for gathering participant-specific data for developing and evaluating interventions, only a few studies in the literature describe the use of personas and scenarios for the definition of novel health care interventions [17,18]. Most have focused on specific, mostly technology-driven, components of the larger system. None demonstrated use of personas and scenarios to design a large system that integrates clinical quality-improvement and research—a learning health system.

### Utility

Deriving dyads of personas based upon interviews with pediatric patients and their parents proved valuable in at least 2 ways. Interview data could be cross-checked with the other family members, helping to clarify dates, diagnostics, treatments, etc, particularly for those events that happened years in the past in the life of a child. Equally important, the success of pediatric medicine can hinge on identifying the underlying values of patients and parents, the qualities of their interactions, and ways for improving communication among all parties. Employing research-based personas and scenarios also helped design of this new health care model whose explicit intent is to level the uneven “authority gradient” common to clinician-patient relationships by providing patients novel means to collaborate more fully in treatment and self-care alternatives [29].

### Limitations

Several limitations of this study should be noted. While the small sample size raises questions of the validity of our results, previous work in this field suggests that a relatively small number of immersive qualitative interviews and observations reveals behavioral patterns necessary for system design, provided that the sample pool represents a range of likely potential participants [13]. This is similar to the concept of theoretical saturation in qualitative research. Moreover, patients, families, clinicians, and researchers who reviewed the resulting personas judged them as valid and useful. The goal of persona development is not to comprehensively portray all potential system participants, but rather to represent distinct types of participants so that a variety of thematic continua can be incorporated into the design. This yields designs that can work for a variety of potential participants represented by those personas.

Another limitation is that it is unlikely health care researchers will find success with these methods unless a trained and experienced interaction designer plays a key role during the initial research and synthesis phases and the first handful of intervention designs. If the work is well documented and other professionals take part in those activities, then those other professionals should be (and indeed in the case of C3N were) able to evolve the persona/scenario set and design/study of future interventions. In other words, GDD methods do not make somebody into a designer; rather, they provide ways for researchers and designers to become more effective teammates.

### Conclusions

Increasing calls for a learning health system highlight the urgent need to translate this concept into reality. New personal health technologies, growing data repositories, and innovative analytic approaches yield clues to some tools that could be useful in creating a learning health system. Specific technologies, tools, and methods, however, are unlikely to form a cohesive human system absent a deliberative design process. Meanwhile, health care interventions are, for the most part, developed and designed by medical or public health researchers with limited, if any, software design experience or expertise. Methods developed for product design and marketing are now used widely in industries outside health care in order to gain a better understanding of users’ attitudes, beliefs, behaviors, and values and to incorporate this information into the design of products and services prior to developing and diffusing them into the marketplace.

We found GDD helpful in elucidating the human context into which novel approaches could be introduced. Moreover, with its emphasis on the human scale of software-powered systems, GDD yielded actionable insights that could be used by designers, software developers, database engineers, physicians, nurses, and patients, and patient advocates alike to make C3N into a learning health care network that simultaneously improves care, promulgates novel clinical and self-care interventions, and discovery.

## Multimedia Appendix 1

Other personas.

## Multimedia Appendix 2

Example scenario and design screenshots.

## Abbreviations

CCHMC: Cincinnati Children’s Hospital Medical Center
C3N: collaborative chronic care network
GDD: goal-directed design
HCI: human-computer interaction
IBD: inflammatory bowel disease
IOM: Institute of Medicine
IRB: institutional review board
UC: ulcerative colitis
UCD: user-centered design

## References

[1] Olsen L, Aisner D, McGinnis JM (2007). The Learning Healthcare System: Workshop Summary.

[2] Greene SM, Reid RJ, Larson EB (2012). Implementing the learning health system: from concept to action. Ann Intern Med.

[3] Friedman C, Rigby M (2013). Conceptualising and creating a global learning health system. Int J Med Inform.

[4] Hogarth M, Hajopoulos K, Young M, Cowles N, Churin J, Hornthal B, Esserman L (2010). The Communication and Care Plan: a novel approach to patient-centered clinical information systems. J Biomed Inform.

[5] Fox S (2013). After Dr Google: peer-to-peer health care. Pediatrics.

[6] Johnson CM, Johnson TR, Zhang J (2005). A user-centered framework for redesigning health care interfaces. J Biomed Inform.

[7] Kinzie MB, Cohn WF, Julian MF, Knaus WA (2002). A user-centered model for web site design: needs assessment, user interface design, and rapid prototyping. J Am Med Inform Assoc.

[8] Martikainen S, Ikävalko P, Korpela M (2010). Participatory interaction design in user requirements specification in healthcare. Stud Health Technol Inform.

[9] Martikainen S, Viitanen J, Korpela M, Lääveri T (2012). Physicians' experiences of participation in healthcare IT development in Finland: willing but not able. Int J Med Inform.

[10] Taylor HA, Sullivan D, Mullen C, Johnson CM (2011). Implementation of a user-centered framework in the development of a Web-based health information database and call center. J Biomed Inform.

[11] Rosson MB, Carroll JM (2002). Usability Engineering: Scenario-Based Development of Human Computer Interaction.

[12] Kano N, Seraku N, Takahashi F, Tsuji S (1984). Attractive quality and must-be quality. J Japan Soc Qual Control.

[13] Cooper A (1999). The Inmates Are Running the Asylum.

[14] Carroll J, Soegaard M, Dam RF (2013). Human computer interaction-brief intro. The Encyclopedia of Human-Computer Interaction, 2nd edition.

[15] Norman D, Draper SW (1986). User Centered System Design: New Perspectives on Human-Computer Interaction.

[16] Vredenburg K, Mao J, Smith P, Carey T (2002). A Survey of User-Centered Design Practice.

[17] Koch S, Sheeren A, Staggers N (2009). Using personas and prototypes to define nurses requirements for a novel patient monitoring display.

[18] Kashfi H (2010). Applying a user centered design methodology in a clinical context. Stud Health Technol Inform.

[19] Lerouge C, Ma J, Sneha S, Tolle K (2011). User profiles and personas in the design and development of consumer health technologies. Int J Med Inform.

[20] D'Alessandro DM, Dosa NP (2001). Empowering children and families with information technology. Arch Pediatr Adolesc Med.

[21] Roehrer E, Cummings E, Ellis L, Turner P (2011). The role of user-centred design within online community development. Stud Health Technol Inform.

[22] Holtzblatt K, Beyer H (1993). Making customer-centered design work for teams. Commun. ACM.

[23] Pruitt J, Grudin J (2003). Personas: practice and theory. Proceeding DUX '03.

[24] ImproveCareNow network.

[25] Crandall W, Kappelman MD, Colletti RB, Leibowitz I, Grunow JE, Ali S, Baron HI, Berman JH, Boyle B, Cohen S, del Rosario F, Denson LA, Duffy L, Integlia MJ, Kim SC, Milov D, Patel AS, Schoen BT, Walkiewicz D, Margolis P (2011). ImproveCareNow: the development of a pediatric inflammatory bowel disease improvement network. Inflamm Bowel Dis.

[26] Sollecito WA, Margolis PA, Miles PV, Perelman R, Colletti RB, McLaughlin CP, Kaluzny AD (2006). Quality in pediatric subspecialty care. Continuous Quality Improvement in Health Care: Theory, Implementations, and Applications.

[27] Crandall WV, Margolis PA, Kappelman MD, King EC, Pratt JM, Boyle BM, Duffy LF, Grunow JE, Kim SC, Leibowitz I, Schoen BT, Colletti RB, ImproveCareNow Collaborative (2012). Improved outcomes in a quality improvement collaborative for pediatric inflammatory bowel disease. Pediatrics.

[28] Patton MQ (1990). Qualitative Evaluation and Research Methods, 2nd edition.

[29] Wetzel AP, Dow AW, Mazmanian PE (2012). Patient safety attitudes and behaviors of graduating medical students. Eval Health Prof.

