# Supplementary material for: Using Goal-Directed Design to Create a Novel System for Improving Chronic Illness Care
Source: JMIR Res Protoc. 2013 Oct 29;2(2):e43. doi: 10.2196/resprot.2749 (PMC3841377; doi:10.2196/resprot.2749)
Supplement: Supplementary file 1 [file resprot_v2i2e43_app1.pdf]

Multimedia Appendix 1 – Illustrative Personas

Persona	Goals	Characteristics
Orleans - Patient<br>• Age 13<br>• African-American<br>• Diagnosed 2 years ago with ulcerative colitis (UC) and autoimmune hepatitis	• Control symptoms<br>• Be understood<br>**Illustrative quote:**<br>*"I like to play video games."*	• Still getting her bearings. Flares are frequent. Often on prednisone, which makes her look and feel unusual.<br>• Struggles with dad on how to care for herself. Has to take many pills that don't always seem to work. Recently diagnosed with depression.<br>• Hard to watch what she eats, particularly when she's with other kids. Socially isolated, repeatedly absent from school, trusts only a few friends. Tries to stay active, practices yoga, plays basketball when she's feeling well. Uses the Internet for playing games, downloading music, email, MySpace. Leaves the Internet IBD research to her dad.
Floyd Jackson – Orleans' Dad<br>Age 38<br>Married<br>3 children<br>Self-employed auto repair shop owner.	• Make the right choices.<br>• Keep track of it all.<br>• Find a healing community.<br>• Maintain financial stability.<br>**Illustrative quote:**<br>*"It would be good for her if she knew someone else with ulcerative colitis."*	• Has no experience with chronic disease. Orlean's primary caretaker: makes appointments, picks up prescriptions, communicates with the nurse. Is concerned about paying for Orleans' ongoing treatment, especially if she gets worse and his wife has to stop working to care for her.<br>• Skeptical about medications, has heard about some long-term side effects. Is interested in non-pharmaceutical therapies. Seeks advice from many sources, including friends and websites.<br>• Worried that Orleans is becoming irritable and solitary and that she is depressed.
Adam - Shadow Patient*<br>• Age 19<br>• Diagnosed with UC 2 years ago	• Feel better<br>• Focus on life outside the disease<br>• Avoid hassles	• Hid symptoms until pain was unbearable and he couldn't make excuses about being in the bathroom so often. Finally told mom, she took him to the Emergency Room, he was admitted and diagnosed with UC.<br>• Prednisone didn't make him feel much better, made him weak, effected his

		exercise. Had insomnia, making it difficult to study. He's been on several medications; he doesn't trust pills and sometimes skips taking them. He finds it difficult to know if the pills aren't working or he's still sick because he's not fully adherent.<br>• Started college, doctor was worried about the transition. Maintained continuity by staying with his current doctor. Wanted to put him on Remicade to reduce the number of pills. Adam canceled appointment and hasn't rescheduled.
Dr. Sandy Roan - IBD Specialist	• Create perfect care program<br>• Build trust with families<br>• Catch kids before they fall through cracks<br>• Stay engaged with other doctors<br>**Illustrative Quote:** *"If my patients are going to get better, I need to track all aspects of their conditions."*	• Works at gastroenterology clinic. Every new IBD case is a puzzle; each patient seems to need a custom-fit treatment, she's challenged and motivated.<br>• Collaborates closely with nurse, Veronica; focuses on data collection and management; stays engaged with her GI colleagues so she is aware of new research.
Veronica Mayfield – IBD nurse	• Not let patients slip through the cracks<br>• Empower patients and families<br>• Provide excellent care<br>• Make a difference<br>**Illustrative Quote:** *"Tracking data can be a hassle, but I can see it working for my*	• Likes pediatric GI because she can educate young patients and form long-lasting relationships with them. Takes patient's vitals and histories and handles patient follow-up.<br>• Veronica and Sandy have been working on a new data collection and tracking system. It's a lot of work, but Veronica can see the data showing an increase in remission rates.

	patients."	
Dr. Kapoor - Research Psychologist	• Improve pediatric GI care<br>• Encourage communication among researchers<br>• Find easy ways to share data<br>•To disseminate his research to a wide audience<br>*"Give me the data and the data behind the data and I can tell you what went on."*	• Wants to work with children, understand their needs, improve communication, identify ways to improve care delivery, and understand how parent behaviors can affect the child's understanding and attitude towards disease, adherence, and long-term outlook.<br>• Wants to share information and research in real-time to improve research design and catch errors early, get academic credit for his work, and reach people who cannot afford the travel and expenses for care and who may live in areas with limited access to latest information/technology.

* A crucial recommendation stakeholders made when reviewing the personas led to addition of a shadow persona, who represents patients who fall through the cracks, but must be taken into account when designing a system intended to help patients better care for themselves. This persona was defined by his absence in the interview set, but was filled out by inference, based upon input from clinicians.
