# Supplementary material for: Using Goal-Directed Design to Create a Novel System for Improving Chronic Illness Care
Source: JMIR Res Protoc. 2013 Oct 29;2(2):e43. doi: 10.2196/resprot.2749 (PMC3841377; doi:10.2196/resprot.2749)

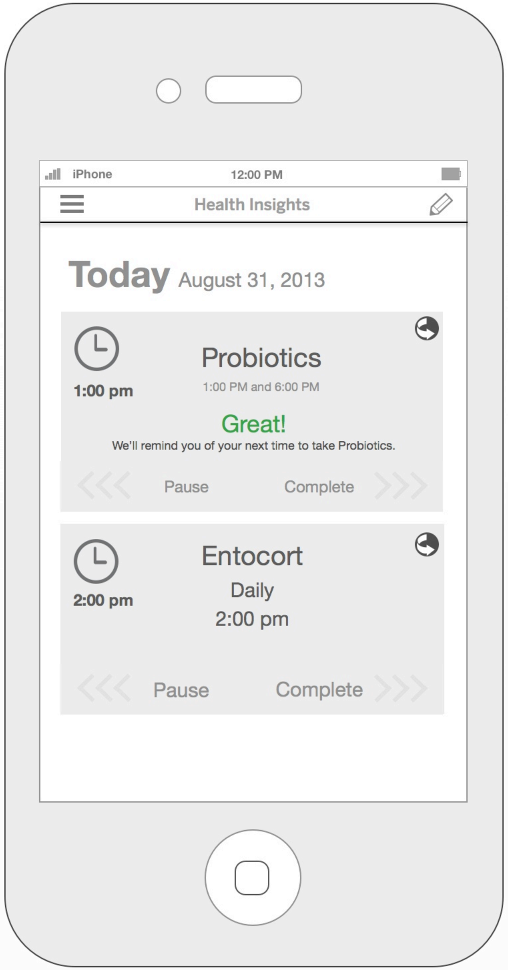

Dr. Roan introduces a new health-assistant app to Floyd and Orleans during a visit. She shows them a demo and asks whether they would like to participate.

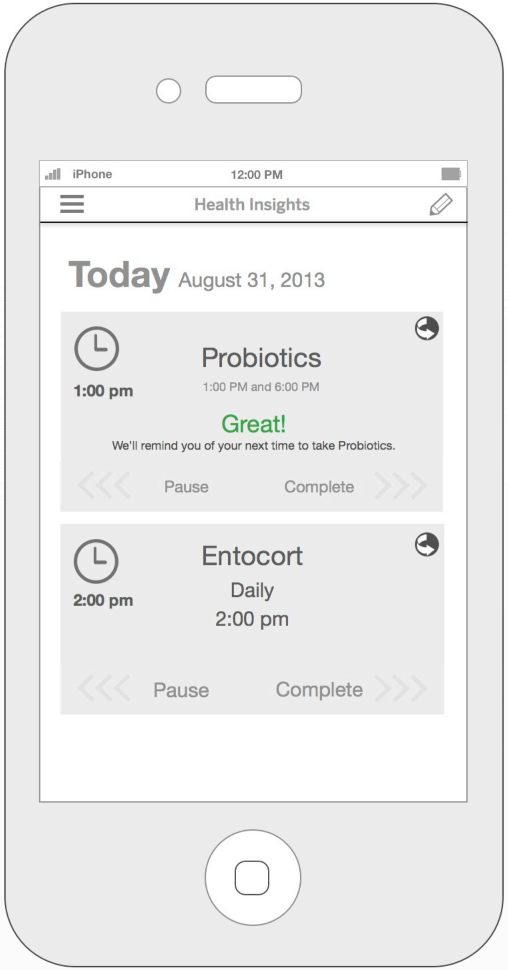

Orleans is reluctant to take on the responsibility, but Floyd sees that it could be very helpful in keeping track of everything he has to do for Orleans and her Ulcerative Colitis.

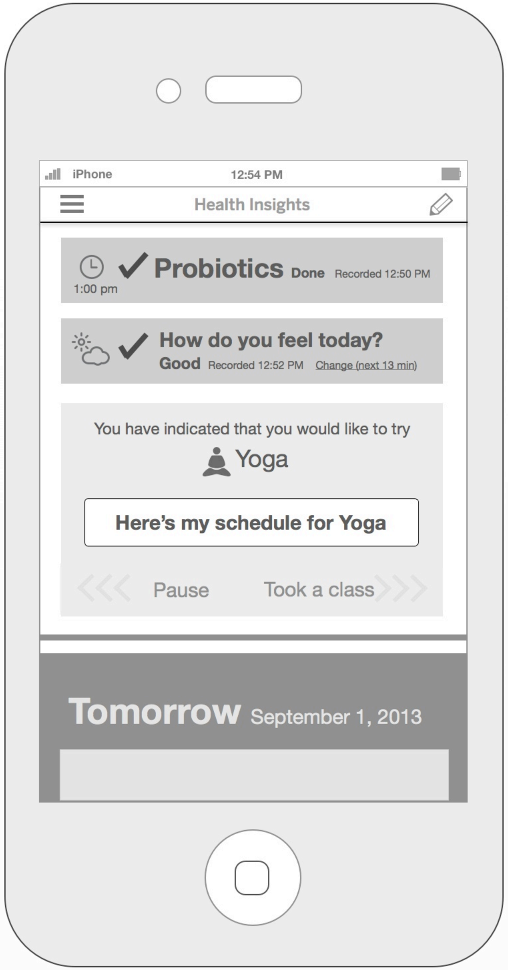

Floyd starts it up the next day. He sees the activities they talked about with Dr. Roan: probiotics, medication, yoga, as well as a survey that asks him how Orleans feels. Each activity is represented by a card.

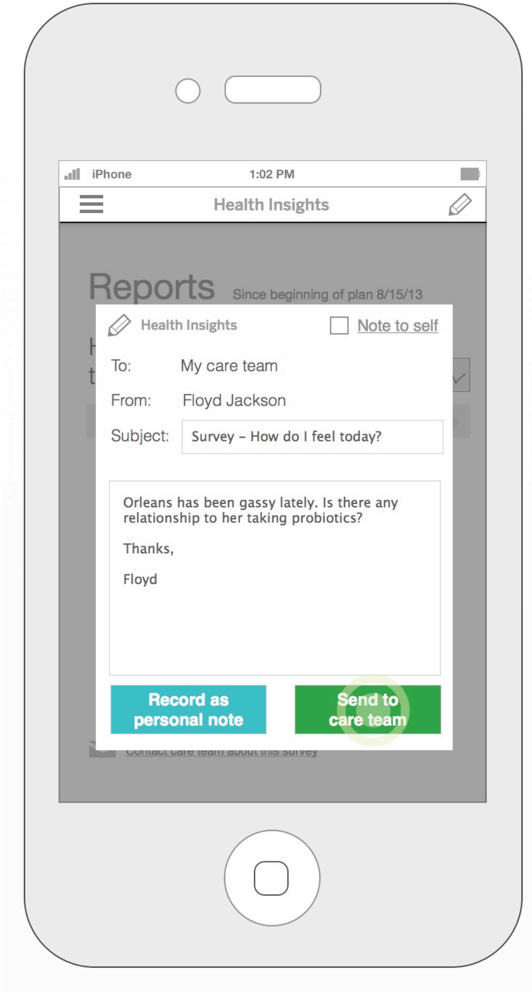

Floyd sends a question about Orleans' probiotics to the care team through the app.

Dr. Roan opens Health Insights on her iPad and does a quick scan of her active patients.

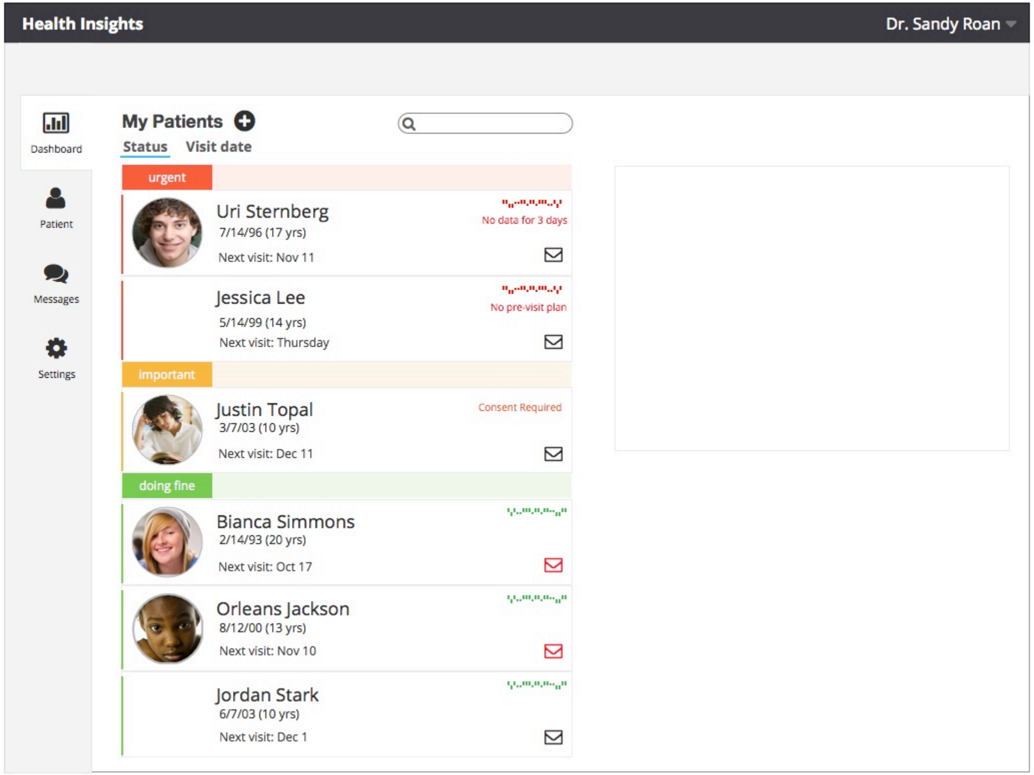

Dr. Roan sees that there're a few patients who have questions and she answers them within the secure environment of the app.

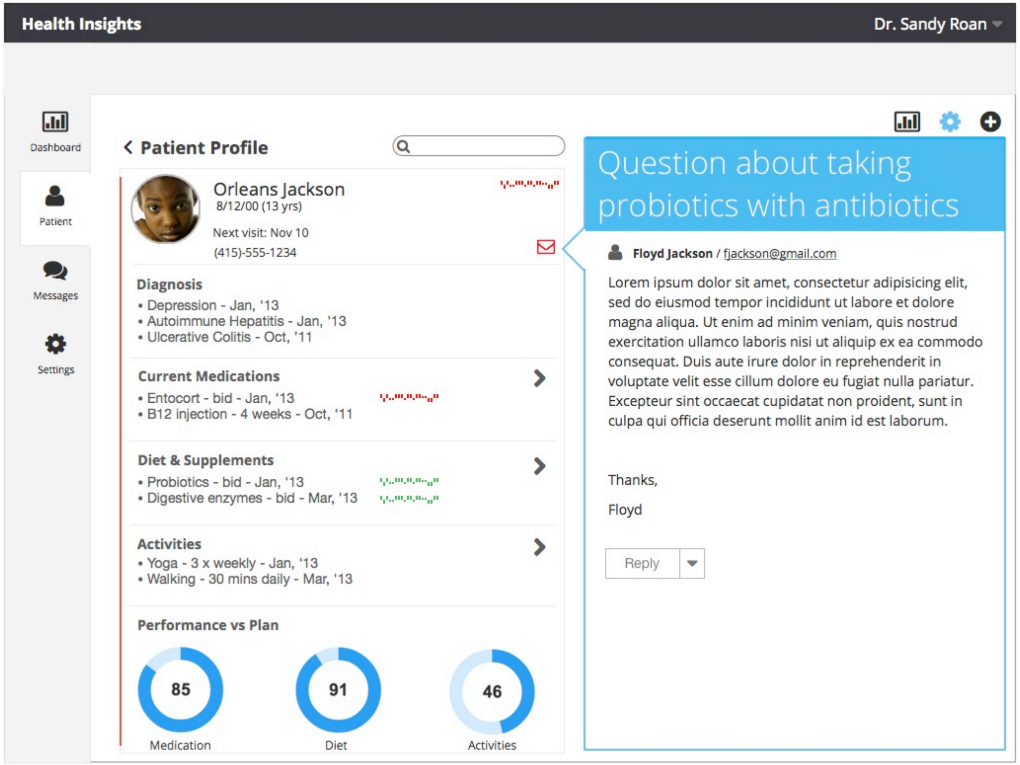

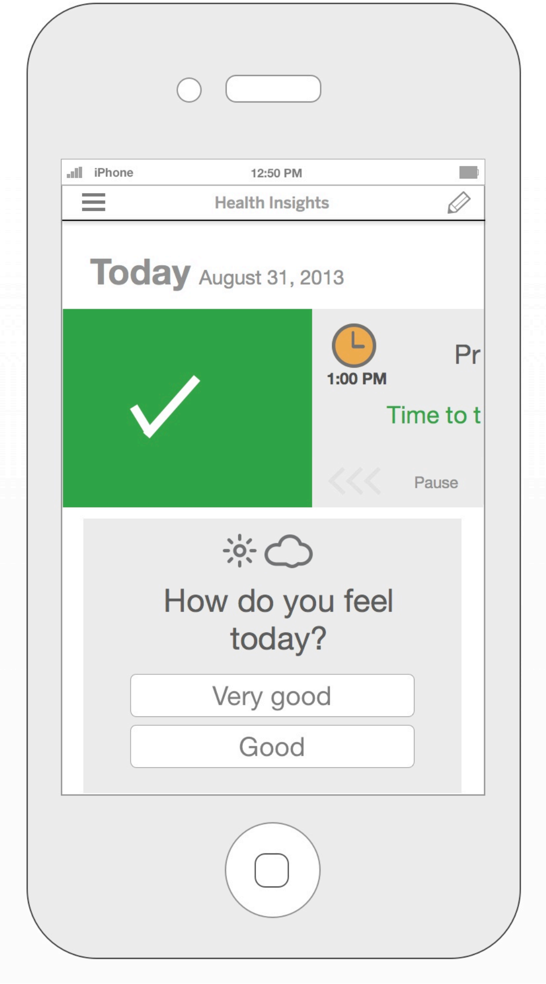

The next day, Floyd swipes one of the plan activity cards to indicate that Orleans has taken her medication.

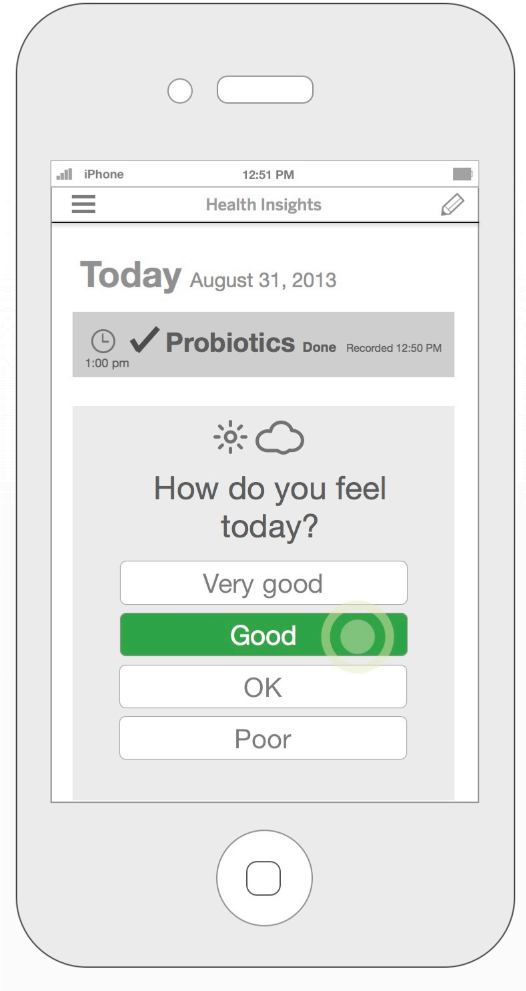

He also answers a survey question about Orleans that he gets every morning.

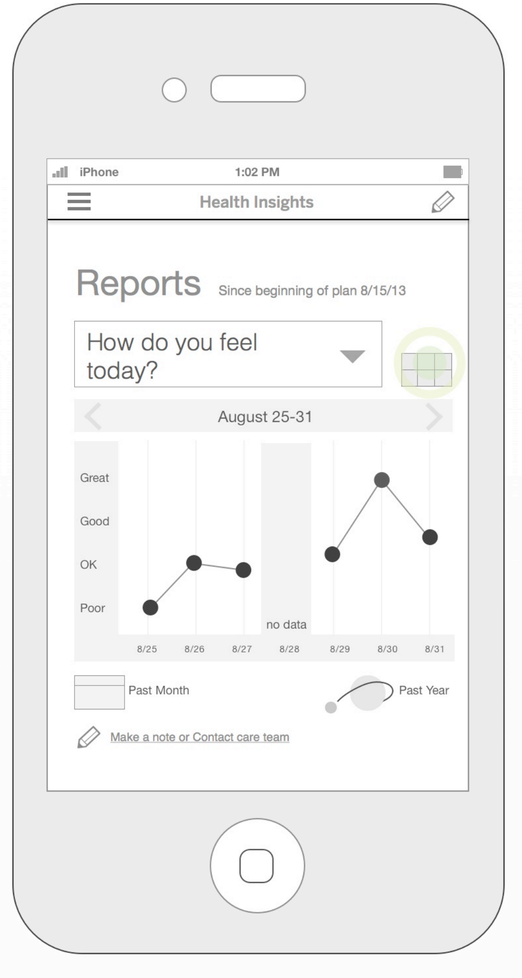

At the end of the week, Floyd wants to see a graph with all the responses of the week. Looks like it’s been an up and down week, which would explain Orleans’ moodiness.

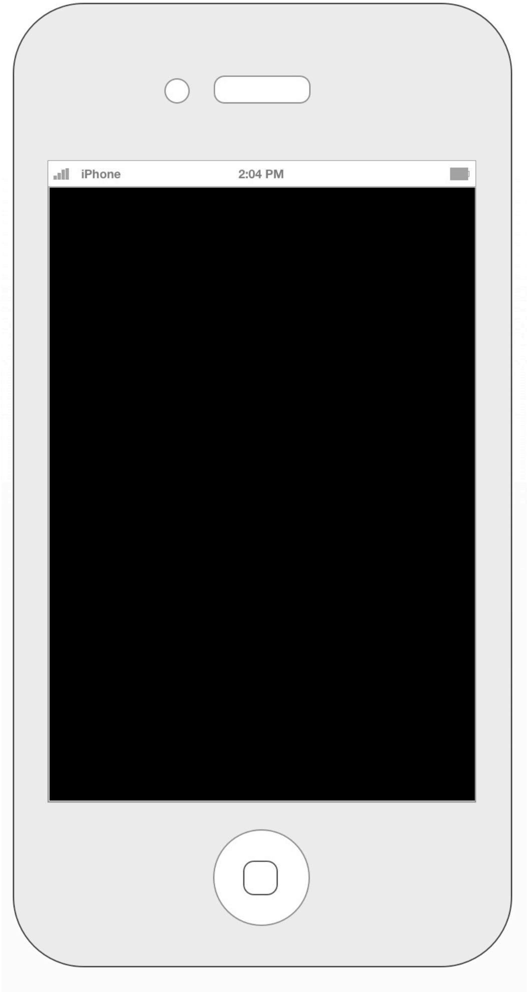

After a few days, Floyd gets really busy with other things, and neglects the app for four days.

Dr. Roan launches Health Insights on her iPad and does a quick scan of her patients. Orleans' account is at the top of the list, because there has been no activity for 4 days.

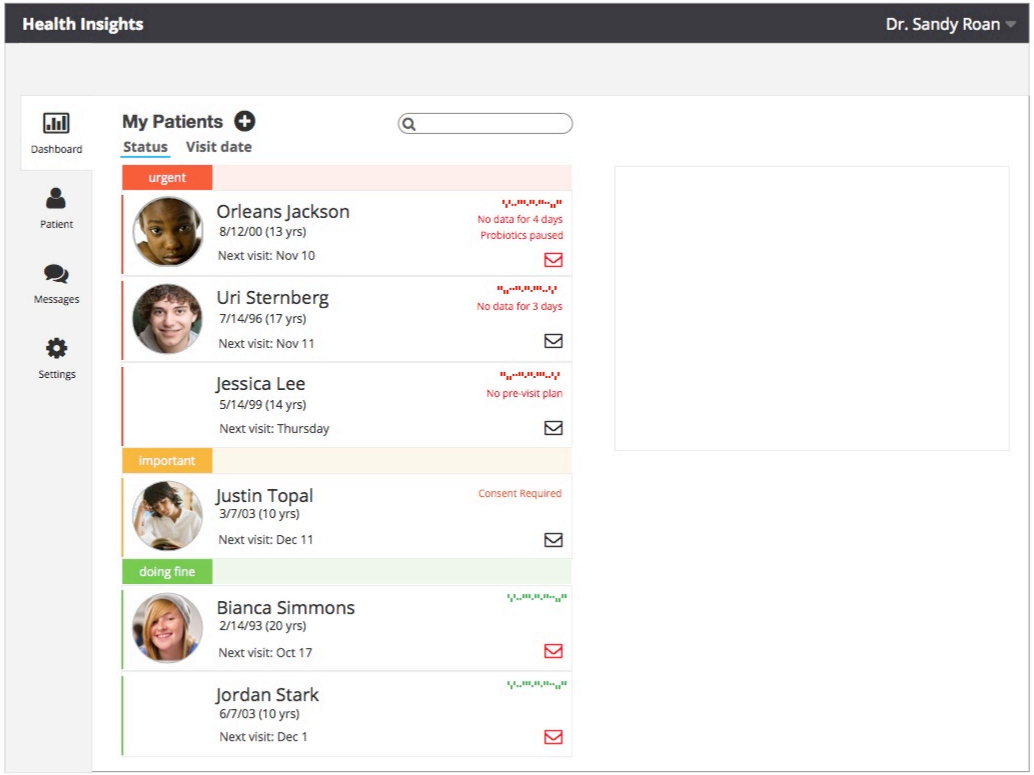

Dr. Roan asks her nurse to call Floyd to check up on Orleans.

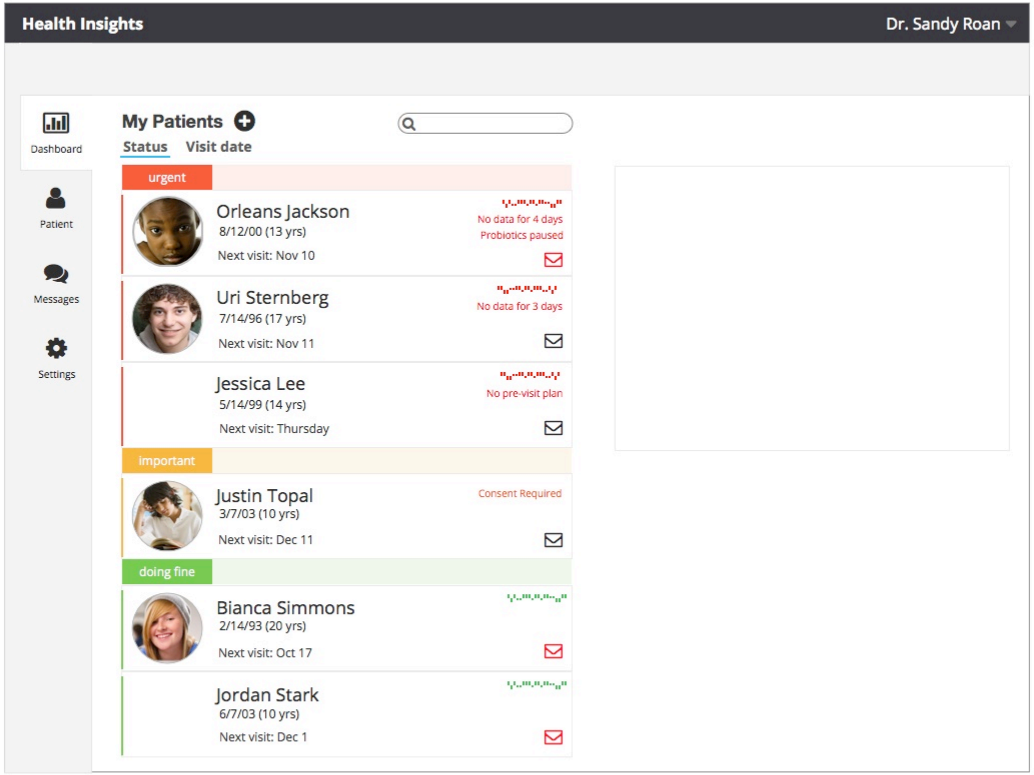

Supplement: Supplementary file 2 [file resprot_v2i2e43_app2.pdf]
